# Antibiotic therapy, supportive treatment and management of immunomodulation-inflammation response in community acquired pneumonia: review of recommendations

**DOI:** 10.1186/s40248-017-0106-3

**Published:** 2017-10-05

**Authors:** Marco Mantero, Paolo Tarsia, Andrea Gramegna, Sonia Henchi, Nicolò Vanoni, Marta Di Pasquale

**Affiliations:** Department of Pathophysiology and Transplantation, Università degli Studi di Milano, Internal Medicine Department, Respiratory Unit and Regional Adult Cystic Fibrosis Center, IRCCS Fondazione Cà Granda Ospedale Maggiore Policlinico, Via Francesco Sforza, 35 Milan, Italy

**Keywords:** Acute respiratory failure, Antibiotic therapy, Community acquired pneumonia, Duration of therapy, Inflammatory response

## Abstract

Community-acquired pneumonia is a common and serious disease, with high rates of morbidity and mortality. Management and treatment of community-acquired pneumonia are described in three main documents: the 2007 American Thoracic Society guidelines, the 2011 European Respiratory Society guidelines, and the 2009 British Thoracic Society guidelines, updated by the NICE in 2015. Despite the validity of current guidelines in improving prognosis and management of patients with community-acquired pneumonia, not all recommendations have high levels of evidence and there are still some controversial issues. In particular, there are some areas of low evidence such as the efficacy of an antibiotic molecule or scheme in patients with same risk factors; duration of antibiotic treatment, supportive therapy for acute respiratory failure and immunomodulation molecules.

This review will summarize the main recommendations with high level of evidence and discuss the recommendations with lower evidence, analyzing the studies published after the guidelines’ release.

## Background

Community-acquired pneumonia (CAP) is a common and serious disease, with high rates of morbidity and mortality. It represents the third leading cause of death worldwide and the sixth in United States of America (USA), affecting more than 4 million adults. CAP is accountable for approximately 1 million hospital admissions with a highly significant impact on health care resources [1]; in the USA the costs amount to more than $20 billion in direct health care annually [2].

Management and treatment of CAP are described in three main documents: the 2007 American Thoracic Society (ATS) guidelines, the 2011 European Respiratory Society (ERS) guidelines, and the 2009 British Thoracic Society (BTS) guidelines, updated by the NICE in 2015 [3–5]. The efficacy of these guidelines in improving different outcomes, such as reduction of unnecessary hospitalizations and readmissions, length of hospital stay, cost and mortality, has been established in different contests [6, 7].

Despite the validity of current guidelines in improving prognosis and management of patients with CAP, not all recommendations have high levels of evidence and there are still some controversial issues. This review will summarize the main recommendations with high level of evidence shared by the main guidelines, and discuss the recommendations with lower evidence, analyzing the studies published after the guidelines’ release.

## Summary of strong recommendations for the treatment of CAP

Below are summarized the recommendations with stronger evidence divided into three main categories: antibiotic therapy, supportive treatment and management of immunomodulation-inflammation response.

1. Antibiotic therapy:
Choice of antibiotic
    The choice of empirical treatment should ensure a spectrum of action with a high probability of covering the germs responsible for pneumonia, and at the same time avoid antibiotics overuse.
    The recommendations for antibiotic choice are different based on the severity of the disease, distinguishing an antibiotic scheme for outpatients, for in-patients and for patients hospitalized in intensive care units (ICU). The recommendations in the various guidelines have a sufficient degree of overlap, although the reported levels of evidence are different (Table 1), highlighting the need for further studies in this area [3–5].

**Table 1:** Empirical antibiotic therapy scheme for Community Acquired Pneumonia in the main international guidelines

	Outpatients		Inpatients	ICU-patients	
ICDS/ATS 2007 Guideline	Previously Healthy	Macrolide Doxycycline	Respiratory Fluoroquinolones Or β-Lactam plus macrolide	No pseudomonas risk factors	Β-Lactam (Cefotaxime, ceftriaxone or ampicillin-sulbactam) plus azithromycin or Respiratory fluoroquinolones. For penicillin-allergic patients, a respiratory fluoroquinolones and aztreonam.
	Comorbidities	Fluoroquinolones or βLactam plus macrolide		Risk factor for P aeruginosa	Antipneumococcal, antipseudomonal β lactam (piperacillin-tazobactam, cefepime, imipenem or meropenem) plus ciprofloxacin or levofloxacin (750 mg) Or plus aminoglycoside and azithroycin
BTS 2009 (Update NICE 2015)	Amoxicillin 500 mg TID OR Clarithromycin (Alternative if Hypersensitive)		Amoxicillin and Macrolide OR Macrolide (Alternative if Hypersensitive) OR Doxycicline OR Levofloxacin or moxifloxacin	Broad spectrum β lactamase antibiotic plus macrolide Second or third generation cephalosporin, if hypersensitivity to β lactam, plus clarithromycin	
ERS/ESCMI 2011	Amoxicillin or Tetracycline Tetracycline or macrolide Alternative Levofloxacin or moxifloxacin (Alternative if hypersensitivity in countries with high incidence of macrolide resistant pneumococcus)		Aminopenicillin plus macrolide Aminopenicillin/β lactamase inhibitor plus macrolide Non antipseudomonas cephalosporin plus macrolide Levofloxacin Moxifloxacin Penicillin G plus macrolide	No pseudomonas risk factors	Non antipseudomonal cephalosporin III plus macrolide Or Moxifloxacine or levofloxacin plus non antipseudomonas cephalosporin III
				Risk factor for P aeruginosa	Antipseudomonal cephalosporin or acylureidopenicillin/βlactamase inhibitor or CarbapenemPlus Ciprofloxacin or Plus Macrolide plus Aminoglycoside

ICDS, Infectious Diseases Society of America; *ATS* American Thoracic Society, *BTS* British Thoracic Society, *NICE* National Institute for Health and Care Excellence, *ERS* European Respiratory Society, *ESCMI* European Society for Clinical Microbiology and Infectious Disease
Route of administration
    Antibiotics should be administered by oral route for outpatients. For inpatients, endo-venous treatment should be switched to oral administration as clinical stability is obtained [3–5].
Monitoring
    Monitoring of pneumonia should be conducted using simple clinical criteria (fever, respiratory rate, hemodynamic parameters) and biomarkers of inflammation such as C-reactive protein (CRP) or procalcitonin (PCT) [3].
    These parameters are fundamental to define clinical stability and, therefore, guide switch to oral antibiotic therapy.
Starting treatment
    Antibiotic treatment should be started within 1 h after the diagnosis of pneumonia in case of septic shock, because this reduces mortality, while the recommendations in the other categories of patients have a low level of evidence [3, 4].
Duration of the treatment
    There are not specific recommendations regarding the proper duration of antibiotic treatment [3–5].
2. Supportive treatment:
  Supportive care is essential to ensure stability of vital functions altered by the acute condition and to prevent complications related to loss of function. Main recommendations from different guideline are summarized in Table 2.
3. Management of immunomodulation-inflammation response
  There are strong recommendations against routine use of steroids, even in severe CAP, or the use of colony stimulating granulocyte [3, 5]. Despite these recommendations, many recently published studies showed great interest on this subject [8–10].

**Table 2:** Supportive care for patients with pneumonia

Respiratory failure	Mild	Oxygen therapy
	Severe	Invasive ventilation with protective low tidal volume (6 ml/kg) if bilateral infiltrate or ARDS
	Severe	NIV contraindicated in the majority could be used as personized option
Prevention of DVT and pulmonary throboembolism	In patients with reduced mobility	LMWH or heparin
Prevention of disabilities		Early mobilization from the day after the onset of the symptoms

*ARDS* Acute Respiratory Distress Syndrome, *NIV* Non Invasive Ventilation, *LMWE* Low Molecular Weight Heparin

## Recommendations with lower evidence and analysis of studies published after guideline release

1. Antibiotic Therapy
Choice of antibiotic
    The choice of the empirical therapy scheme should be the best possible for the treatment of pneumonia and individualized for each patient, in terms of efficacy of the antibiotic (single agent or combination treatment) and ability to detect the presence of germs with particular profiles of resistance.
    Recommendations for the choice of antibiotics differ among guidelines and among the various sub classes of patients: outpatients, inpatients, intensive care unit (ICU)-patients. In particular, the body of evidence indicating the superiority of β-lactam and combination of β-lactam with a macrolide are based on cohort studies and observational studies [11], but studies with more adequate design are needed.
    Two randomized controlled trials (RCTs) have been recently published addressing this topic [12, 13]. The first evaluates the equivalence of efficacy of beta-lactam antibiotic alone vs beta lactam plus macrolide or vs levofloxacin alone in the treatment of hospitalized CAP. Results showed the non-inferiority of beta lactam vs the actual recommended scheme. Nevertheless, this study is unlikely to impact on clinical practice because a great number of recruited patients did not meet criteria for hospitalization, and guidelines already suggest the use of beta lactam monotherapy for mild and moderate CAP.
    The second RCT included moderate and severe patients, and again compared beta-lactam monotherapy with combination treatment [13]. Results showed a trend toward superiority of the combination of beta-lactam and macrolide compared to beta-lactam monotherapy in achieving clinical stability. A greater effect was found in patients with more severe forms of pneumonia and in patients whose infection was sustained by “atypical” pathogens. The results of the study are not conclusive but seem to confirm the clinical approach recommended by the guidelines [12, 13].
    A systematic review evaluated studies that compared the efficacy of treatment with fluoroquinolones versus combination therapy with macrolides and beta-lactams in adult patients hospitalized with CAP. Seventeen studies were included (16,684 patients) but no randomized controlled study was identified and the body of evidence had very low quality. Therefore, recommendations cannot be made in favor or against the two different regimens of treatment [14].
    New antibiotics could increase the efficacy of pneumonia treatment in comparison with the molecules and combinations available today. One of these is Solitromicine, a fourth-generation macrolide, that demonstrated non-inferiority compared to Moxifloxacin in a phase III study that enrolled patients from North and Latin America, Europe and South Africa, suggesting a possible role of new macrolide antibiotics in the treatment of CAP [15].
    The presence of multi drug resistant pathogens among patients with CAP is concerning, as it represents a possible cause of treatment failure and worse prognosis.
    To address this topic the definition of health-care associated pneumonia (HCAP) was developed in 2005. The proposed definitions were then found not to be fully applicable due to a lack of scientific evidence and for the tendency towards large-spectrum antibiotic overuse [16]. Models based on clinical risk factors and scores were subsequently proposed to identify patients with CAP but at risk for infection with multidrug resistance germs [17–21].
    Models to detect multidrug resistance bacteria have been proposed by the studies of Shorr and Aliberti [17, 18]. Shorr score is thus composed: 4 points if recent hospitalization, 3 points if presenting from long term facilities, 2 points if on hemodyalisis, 1 point if admitted to the ICU within 24 h of evaluation in the emergency department. The Aliberti score attributes 5 points in presence of chronic renal failure, 4 points if hospitalization for more than 2 days in the preceding 90 days, 3 points if resident in a nursing home or extended care facility, 0.5 points if one of the following are present: cerebrovascular disease, diabetes, chronic obstructive pulmonary disease (COPD), antimicrobial therapy in the preceding 90 days, immunosuppression, home care wound care, home infusion therapy.
    These models showed a higher efficacy in identify patients with CAP due to multidrug resistant germs compared with the HCAP definition [17, 18].
    The classic risk factors for *Pseudomonas aeruginosa* infections (lung structural abnormalities, recent use of antibiotics or recent hospitalization and use of corticosteroids) do not intercept all patients with CAP due to *Pseudomonas aeruginosa* as showed by a population-based study conducted in the US in which even patients with dementia and cerebrovascular disease had a higher incidence of this pathogen [22]. The use of empirical antibiotics active also against *Pseudomonas aeruginosa* in patients who are then found infected with this germ, improved the prognosis regardless of the presence or absence of risk factors [22] indicating that it is the presence of the pathogen and no other factor that affects the prognosis of these patients.
    Methicillin-resistant *Staphylococcus aureus* (MRSA) has recently been found to be a frequent cause of CAP [23].
    This concern has led to the development of an international, multicenter, point-prevalence study to determine specific risk factors associated with MRSA infection in hospitalised patients with community acquired pneumonia (GLIMP study) [24]. The GLIMP study involved 222 hospitals in 54 countries across 6 continents. The global incidence of MRSA in CAP was about 3%, but with significant regional differences: incidence was >10% in Eastern Europe, China and Brazil, 10.5% in the United States, Northern Europe and Australia, 1–5% in Southern Europe and France. The main risk factors identified by the study were previous MRSA infection, recurring skin infections, and the presence of severe pneumonia. [24]
    The different geographical distribution of resistant bacterial strains must be taken into account when we treat patients empirically. For example, an observational study conducted in Japan showed a greater efficacy of moxifloxacin compared to levofloxacin or Beta lactam in patients with CAP not requiring hospitalization [25]. The result is especially interesting for the Eastern regions of the world, but it is not generalizable and differs from that reported by other similar studies conducted in regions with lower levels of resistance to levofloxacin [26].
    The availability of accurate information about risk factors for infection by specific pathogens and local peculiarity are important to implement local guidelines, in order to change clinical practice and in particular the choice of empirical antibiotics for CAP [27]. An observational study conducted after the introduction of internal guidelines that limited the use of fluoroquinolones and third-generation cephalosporins as empirical therapy for pneumonia, found a decrease in length of stay and hospital mortality, [28] probably due to a better allocation of antibiotic therapy and clinical resources.
Starting treatment
    The recommendation to start antibiotics within 4 h from the diagnosis of pneumonia in the absence of septic shock had low level of evidence; nevertheless a study on the implementation of the bundles of pneumonia management revealed that early administration of antibiotics is one of the more consistent interventions leading to reduced 30-day mortality [30-day mortality IP 22/250 (8.8%) vs. 253/1862 (13.6%), adjusted OR 0.59, 95% CI 0.95 to 0.37, *p* = 0.030] [29], confirming the need of an early start of adequate antibiotic treatment for pneumonia.
Duration of antibiotic therapy
    The majority of available studies that give precise recommendations about the proper duration of antibiotic therapy for pneumonia are applicable in the management of severe pneumonia, especially for patients treated in the Intensive Care Unit (ICU). In this context, the duration of antibiotic treatment can be guided by the trend of procalcitonin levels; antibiotic could be interrupted when PCT levels reach 0.1 ng/ml [30].
    Despite this evidence, there is tendency towards protracted empirical antibiotic therapy in the absence of justifiable criteria, even in the management of ICU patients [31]. This finding indicates how strong is the feeling of security that prolonging antibiotic treatment induces in physicians, as opposed to not prescribing or interrupting antibiotic therapy in the absence of a clear need for these drugs.
    This feeling is even greater in the setting of non-severe pneumonia where consistent data regarding adequate duration of antibiotic therapy are lacking and proper studies are needed. In this contest, a RCT conducted in Spain tried to assess the difference in the effectiveness of short antibiotic treatment (outstanding Antibiotic 48 h after reaching clinical stability) vs. a traditional treatment based on individual medical judgment. The study has the merit of being one of the first prospective studies on this subject to be completed and sustained the use of short course antibiotic therapy, based on equal efficiency but lower rate of side effects or development of bacterial resistance. Unfortunately, the results presented are not applicable in clinical practice yet because of some methodological bias, in particular the choice of a clear primary outcome, the calculation of sample size, as well as the low generalizability due to the extensive use of fluoroquinolones (80% of cases) [32].
    Another recent multicenter, non-inferiority, randomized, controlled trial of hospitalized adult patients with CAP was conducted to evaluate antibiotic duration [33]. Patients who reached clinical stability within 5 days after hospitalization were randomized to a standard vs. individualized antibiotic duration. Unfortunately, the monitoring and safety board committee decided to interrupt the trial because a higher rate of early failure at 30 days was found at the intention-to-treat analysis in patients with de-escalation of therapy based on clinical stability in comparison to the standard group. In addition, authors found protocol violation in 17% of cases, causing a significant difference between intention-to-treat and per protocol analysis. These findings suggest that physicians are more confident in prolonging antibiotic therapy even when clinical stability is reached [31]. Specifically, the authors discussed that time to clinical stability may not be the correct criterion to identify patients who would benefit from individualized therapy as no differences were found between patients who did or did not undergo protocol violation. Moreover, even the 5 days cut off may be incorrect, as the majority of protocol violations occurred when clinical stability was reached between day 4 and day 5, in comparison to patients reaching clinical stability on day 3. The authors underlined that the 3 days cut off may be used for further trials. Finally, 20% of patients refused to participate for concerns regarding discontinuing antibiotic therapy, and only 18% of patients were randomized, limiting the generalizability of the results.
2. Supportive treatment
  Adequacy of empirical antibiotic therapy is essential for a good outcome [34], but it is not sufficient in all cases. In fact, despite an adequate antimicrobial treatment, patients with at least two of the following risk factors 1) hypoalbuminemia (<30 mg / L), 2) need for hospitalization, 3) arterial blood pH <7.35, 4) tachypnea (FR > 30 bpm) and 5) high levels of urea, have a significantly higher 30-day mortality. The early identification of patients who might benefit from an additional supportive therapy is important for a better management and hopefully a better prognosis [35].
  Serum PCT concentration is associated with the risk of requiring invasive ventilation or vasopressors support in adults hospitalized with CAP. The risk rises by 1–2% for each 1 ng/mL increase in PCT when PCT level are less than 10 ng/ml, over this limit the risk remains relatively constant at 22.4%. [36] This finding could be useful when evaluating ICU admission in severe CAP.
Respiratory failure
    The most frequently required supportive care occurs in patients with respiratory failure.
    The studies conducted after the guidelines’ publication evaluated the use of non-invasive respiratory support methods, particularly the use of Helmet Continuous Positive Airway Pressure (CPAP), Non-invasive ventilation (NIV) and high flow nasal cannula oxygen therapy (HFNCO) in the management of severe acute respiratory failure due to pneumonia.
    The use of Helmet-CPAP reduced the risk of meeting predictive criteria for intubation in comparison to oxygen therapy in severe acute respiratory failure due to pneumonia [37] providing a possible tool to avoid treatment with invasive ventilation in these patients.
    Application of NIV in acute respiratory failure due to CAP is more controversial and showed a possible increase in mortality in patients with *de novo*-respiratory failure, probably due to a delay in intubation caused by a prolonged use of NIV. Conversely, in patients with a previous episode of respiratory insufficiency or with acute on chronic respiratory failure, NIV seems to be a safer option [38].
    The use of HFNC in acute respiratory failure does not seem to be superior to traditional oxygen delivery systems or to NIV in reducing the need for intubation. However, in a *post hoc* analysis, patients with more severe respiratory failure (PaO_2_/FiO_2_ < 200) had lower risk of intubation if treated with HFNC compared to traditional oxygen or NIV, indicating a possible role of this technique in this type of patients [39]. Further studies are needed to identify the precise indication of HFNC in CAP.
    Another study gave similar preliminary data about the success of HFNC using the ROX index (Respiratory rate-oxygenation), the ratio of SpO_2_/FiO_2_ to Respiratory rate [40]. Patients with an index greater than 4.88 after 12 h of treatment with HFNC had a significantly different percentage of intubation in comparison to patients with a lower ROX index. Despite some limits, this study identifies one of the first tools specifically applicable to the HFNC technique in the treatment of acute respiratory failure in CAP [40].
    3) Management of immunomodulation-inflammation response
    There has been little progress in the management of CAP since the widespread introduction of antibiotics in the 1950s. Among other causes, it seems that in some patients treatment failure depends on an excessive inflammatory response. On the basis of this hypothesis, different agents with an immunomodulatory activity had been tested in the treatment of CAP.
Macrolides
    Macrolides are used in various fields as immunomodulants, particularly in the treatment of chronic infection with *Pseudomonas aeruginosa* in patients with cystic fibrosis [41] and to a lesser extent in patients with bronchiectasis not caused by cystic fibrosis (NCFBC) [42].
    A retrospective study conducted in patients with pneumonia caused by *Pseudomonas aeruginosa* showed no evidence of a clear benefit in terms of mortality at 30 days in patients treated with macrolides, suggesting that the immunomodulatory role of macrolides in this type of conditions did not modify patients prognosis [43]. However, due to the retrospective design of the study, it is not possible to draw definitive conclusions about the use of macrolides as immunomodulants in the treatment of CAP due to *Pseudomonas aeruginosa*. Future prospective randomized studies are needed to better understand the usefulness of macrolides in this contest.
Immunoglobulins
    The use of immunoglobulins in patients suffering from severe pneumonia is not routinely recommended. The Japanese guidelines indicate immunoglobulins as a possible treatment with a low level of evidence [44]. Nevertheless, a cohort study that involved ICUs in the entire country of Japan provided no new evidence in favor of using immunoglobulins in severe CAP [45]. The use of immunoglobulins in severe CAP is, therefore, not applicable in absence of specific immune response deficits.
Statins
    There are several observational studies that seem to indicate a role of statins in improving the prognosis of patients with pneumonia, but at the moment the possible mechanisms of action are not known and it is not possible to affirm their clinical utility with certainty [46]. A study specifically evaluating neutrophil activation by statins during sepsis will soon be conducted; this study could provide data about their real mechanism of action in infections, thus giving the rational basis for future clinical studies [47].
Corticosteroids
    A series of scientific studies published in the last 10 years and two recent meta analyses showed that the use of corticosteroids in pneumonia may reduce the risk of acute respiratory distress syndrome (ARDS), length of stay (LOS), length of stay in the ICU, risk of clinical failure [8], duration of antibiotic therapy and time to clinical stability [9].
    On the contrary, in other studies patients treated with adjunctive corticosteroid therapy had a higher incidence of hyperglycemia requiring insulin therapy and there was not clear evidence of a reduction in mortality [10, 48].
    There are many unanswered questions on this topic: which type of corticosteroid should be used? Could there be a rebound in the inflammatory response when treatment is stopped? Do all patients benefit from steroid treatment? Which is the better route of administration?
    Some of these questions have only partially been answered. From a *post hoc* analysis, it seems that patients with pneumococcal pneumonia did not benefit from corticosteroids, in terms of reduction of antibiotic treatment duration [49]. This could pose a limit to the indication of steroid treatment in CAP, as *S pneumoniae* is the most frequent pathogen causing pneumonia in the community.
    Corticosteroids may be useful in certain subgroups of patients with pneumonia, but at the moment the use of corticosteroids in pneumonia does not provide better results than the proper application of the guideline recommendations [50].
Pidotimod
    Among immunomodulators, Pidotimod is a synthetic dipeptide molecule endowed with immunomodulatory activity that affects both innate and adaptive immune responses. Previous positive effects on inflammation were obtained in in vitro studies [51, 52]; and subsequently in in vivo studies, where the drug reduced the incidence of upper and lower airways symptoms in children [53], and augmented the influenza specific immune responses in patients with Down syndrome who underwent vaccination [54]. Lastly, a recent multicenter, interventional, prospective study was conducted on adult patients hospitalized with community acquired pneumonia [55]. Patients were randomized to receive either levofloxacin 500 mg bis in die, alone or levofloxacin plus PDT (800 mg, 2 daily doses). Supplemental therapy with pidotimod resulted in an increased antimicrobial and immunomodulatory effect and in a reduction of inflammatory chemokines. Thus, authors suggest that adjunctive therapy with pidotimod could have a positive effect on innate immunity among patients with CAP, but the use of immunomodulators are not recommendable in clinical practice yet.

## Expert opinion

Treatment of CAP is well described by the guidelines now available and its efficacy is demonstrated. However, there are some areas of low evidence, in particular the efficacy of single agent over combination treatment in patients with similar risk factors; duration of antibiotic treatment and supportive treatment. Studies published after the guidelines are interesting but not sufficiently conclusive to modify the above recommendations.

Undoubtedly, further studies are needed to address the duration of therapy issue, in order to acknowledge if an earlier suspension of antimicrobial treatment is viable to all hospitalized patients with CAP and avoid antibiotic overuse. Future studies need to be less strict in selecting criteria, including a more heterogeneous population, resembling real life.

Multi drug resistant organisms (MDRO) are nowadays a challenge for all clinicians, and respiratory tract infections are also included, with more and more cases of MDRO among patients coming from the community. Clinicians are, therefore, concerned by these difficult-to-treat respiratory infections, as the available antimicrobials are actually often ineffective. In recent years, researchers have aimed at developing new molecules, in order to avoid present resistances. There are great expectations regarding novel antibiotics, and studies are awaited to prove their efficacy in community acquired respiratory infections.

Unfortunately, as the majority of novel antibiotics are under development in phases I and II and some in trial phase III, clinicians can currently only count on available molecules. This has originated the search for adjunctive therapies in CAP patients, which can enhance the effect of antibiotics and adjuvate immune response. These molecules could be an alternative strategy to implement the efficacy of standard CAP treatments.

## Abbreviations

CAP: Community Acquired Pneumonia
USA: United States of America
ATS: American Thoracic Society
ERS: European Respiratory Society
BTS: British Thoracic Society
CRP: C-reactive protein
PCT: Procalcitonin
ICU: Intensive care unit
RCT: Randomized controlled trial
HCAP: Health-care associated pneumonia
COPD: Chronic obstructive pulmonary disease
MRSA: Methicillin-resistant *Staphylococcus aureus*
CPAP: Continuous Positive Airway Pressure
NIV: Non-invasive ventilation
HFNCO: High flow nasal cannula oxygen therapy
NCFBC: Bronchiectasis not caused by cystic fibrosis
ARDS: Acute respiratory distress syndrome
LOS: Length of stay
MDRO: Multi drug resistant organisms
ICDS: Infectious Diseases Society of America
NICE: National Institute for Health and Care Excellence
LMWE: Low Molecular Weight Heparin
